# Surgical Resection of a Rapidly Enlarging Endotracheal Schwannoma under Veno-Venous Extracorporeal Membrane Oxygenation Support: A Case Report

**DOI:** 10.70352/scrj.cr.26-0686

**Published:** 2026-10-28

**Authors:** Ryota Yasuda, Yasuaki Kubouchi, Sachiko Yasuda, Hiroshi Ishii, So Futagoishi, Masaya Yamasaki, Yuji Nozaka, Wakako Fujiwara, Shinji Matsui, Yoshiteru Kidokoro, Yugo Tanaka

**Affiliations:** 1 Department of Surgery, Division of General Thoracic Surgery and Breast and Endocrine Surgery, Faculty of Medicine, Tottori University, Yonago, Tottori, Japan; 2 Department of Pathology, Faculty of Medicine, Tottori University, Yonago, Tottori, Japan

**Keywords:** tracheal schwannoma, rapid enlargement, veno-venous extracorporeal membrane oxygenation, tracheal resection

## Abstract

**INTRODUCTION:**

Primary tracheal schwannoma is an extremely rare benign tumor, and despite its typical slow growth, it can cause life-threatening airway obstruction as it enlarges. We report a case of an endotracheal schwannoma that exhibited rapid enlargement during the preoperative waiting period. The tumor was safely resected with veno-venous extracorporeal membrane oxygenation (V-V ECMO) support because of the risk of complete airway obstruction.

**CASE PRESENTATION:**

A 73-year-old woman presented with hemoptysis. CT revealed a broad-based endotracheal tumor measuring 11 × 9 × 14 mm that was located 30 mm proximal to the carina. Bronchoscopic biopsy suggested benign schwannoma. The patient returned with worsening dyspnea and increased cough 2 weeks later. Follow-up CT showed rapid enlargement of the tumor to 17 × 12 × 21 mm, leaving a residual airway diameter of 4.2 mm. Given the risk of ventilation failure during the induction of general anesthesia, V-V ECMO was established while the patient was awake under local anesthesia. After adequate extracorporeal gas exchange was confirmed, general anesthesia was induced, followed by orotracheal intubation. Through a median sternotomy, the cervical and upper mediastinal trachea was exposed. The tumor-bearing tracheal segment was circumferentially resected, and primary end-to-end anastomosis was performed. The postoperative course was uncomplicated, and the patient remained recurrence-free at 12 months after surgery.

**CONCLUSIONS:**

Although benign tracheal schwannomas are generally slow-growing, rapid enlargement can occur and potentially cause life-threatening airway obstruction. In patients with severe tracheal obstruction, planned establishment of V-V ECMO before induction of general anesthesia may provide an additional margin of safety during airway management and tracheal reconstruction.

## Abbreviation

ACT
: activated clotting time
V-V ECMO
: veno-venous extracorporeal membrane oxygenation

## INTRODUCTION

Schwannomas are benign tumors arising from Schwann cells of the nerve sheath. These tumors, which generally grow slowly, can arise at various sites, including the peripheral nerves, posterior mediastinum, spinal nerve roots, and cerebellopontine angle. Primary tracheal schwannomas are extremely rare, accounting for a small proportion of all tracheal tumors.^1)^ We report a case of a primary endotracheal schwannoma that enlarged rapidly during the preoperative waiting period. Because the induction of general anesthesia was considered to carry a risk of complete airway obstruction, V-V ECMO was established while the patient was awake under local anesthesia before general anesthesia was induced. We describe the clinical course and surgical strategy used in this case.

## CASE PRESENTATION

A 73-year-old woman with no significant medical history or smoking history was referred to our hospital because of blood-streaked sputum. She also reported increased sputum production in the supine position. Chest CT revealed a sessile intratracheal mass located 30 mm proximal to the carina that measured 11 × 9 × 14 mm (**Fig. 1A**). Pathological examination of the biopsy specimen suggested benign schwannoma. Complete bronchoscopic removal was considered difficult, and surgical resection was planned (**Fig. 1B**). While awaiting elective surgery, the patient presented with worsening cough and dyspnea. Follow-up chest CT performed 2 weeks after the initial scan revealed rapid enlargement of the tumor to 17 × 12 × 21 mm, leaving a residual airway diameter of 4.2 mm (**Fig. 1C**). Because of the high risk of airway obstruction, the patient was admitted earlier than originally scheduled for close monitoring. Given the risk of ventilation failure during the induction of general anesthesia, V-V ECMO was established while the patient was awake under local anesthesia, with cannulation of the right femoral and right internal jugular veins. The cannulation sites and target ACT of approximately 200 s were determined preoperatively in consultation with the anesthesiology and cardiovascular surgery teams. Before cannulation, 5000 IU of heparin was administered, resulting in an ACT of 210 s at V-V ECMO initiation. During ECMO support, the ACT ranged from 173 to 212 s, and additional heparin was administered as needed to maintain the target ACT, resulting in a total intraoperative dose of 10000 IU. After adequate extracorporeal gas exchange was confirmed, general anesthesia was induced, followed by orotracheal intubation. The tumor was located sufficiently cranial to the carina to permit tracheal resection without carinal reconstruction (**Fig. 1D**). Thus, median sternotomy was selected to provide adequate exposure around the brachiocephalic artery, left brachiocephalic vein, and left common carotid artery. A 3-ring segment of the trachea containing the tumor was resected. After transection of the trachea, cross-field ventilation was initially established through the operative field. Because the tube interfered with the anastomotic procedure, it was removed, and oxygenation was maintained solely with V-V ECMO without ventilation during tracheal reconstruction. The posterior half of the tracheal anastomosis was reconstructed using a running 4-0 polypropylene suture with 5 stitches. An interrupted 4-0 polypropylene anchor suture was placed at each lateral midpoint and tied to the running suture. The anterior half was reconstructed using 7 interrupted 4-0 polypropylene sutures, with each bite incorporating 1 tracheal ring on both the proximal and distal sides (**Fig. 2A**). After completion of the anastomosis, the endotracheal tube was advanced beyond the anastomosis into the distal trachea. Once stable ventilation and oxygenation were confirmed, the patient was successfully weaned from V-V ECMO. The operative time was 267 min, and the estimated blood loss was 1525 mL. The patient received 8 units of red blood cells and 6 units of fresh frozen plasma. She was extubated in the operating room and transferred to the ICU. A cervical collar was applied immediately after surgery to restrict neck extension. Bronchoscopy on POD 9 showed no evidence of anastomotic dehiscence or stenosis (**Fig. 2B**). Intraoperative frozen-section analysis was not performed, and the extent of resection was determined macroscopically. The final pathological diagnosis was benign schwannoma, with an R0 resection and a minimum surgical margin of 0.2 mm. The Ki-67 labeling index was approximately 15% in both the preoperative biopsy specimen and the resected specimen. No features suggestive of malignancy were identified (**Fig. 3A**). Histological examination revealed intratumoral hemorrhage and inflammatory changes, which may have contributed to the rapid enlargement (**Fig. 3B**, **3C**). The tumor arose from the membranous wall of the trachea and exhibited a dumbbell-shaped configuration with both intraluminal and extraluminal components (**Fig. 4**). The postoperative course was uneventful, and the patient was discharged on POD 11. At 12 months postoperatively, she remains well with no evidence of recurrence.

**Fig. 1:**
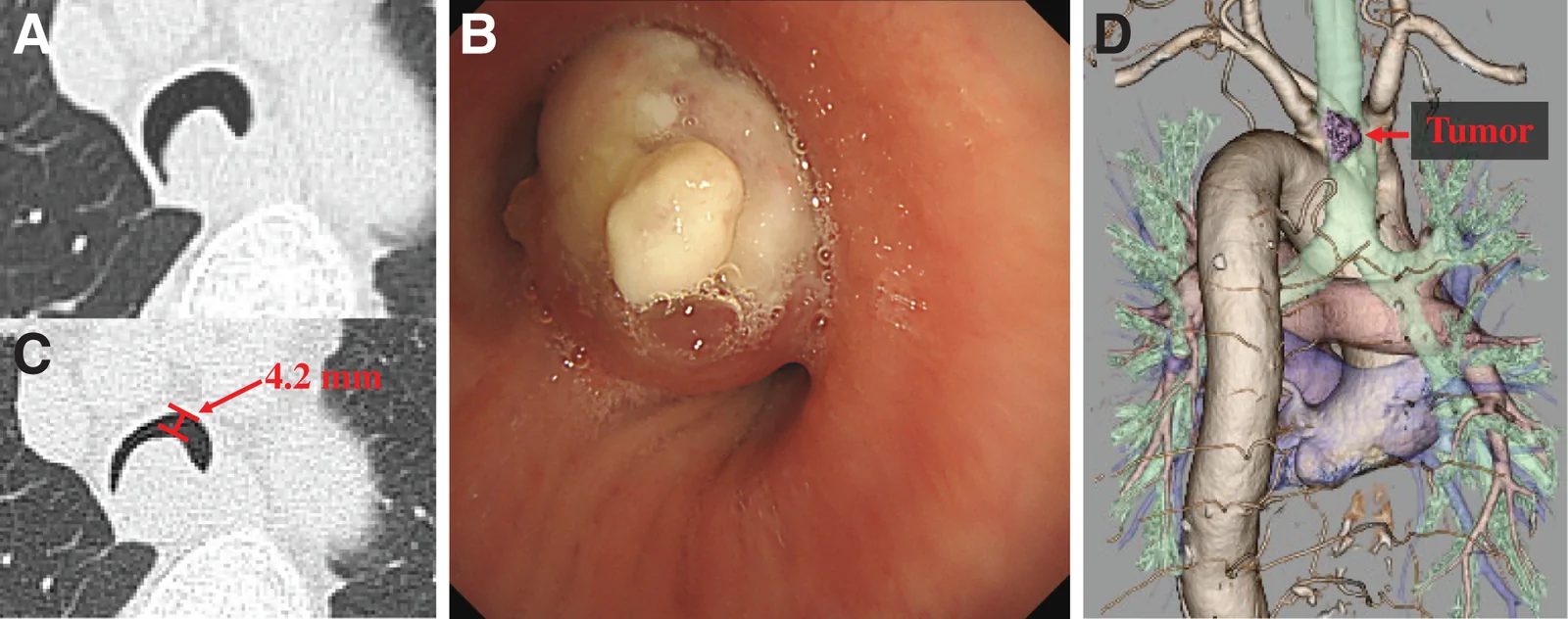
CT and bronchoscopic findings of tracheal schwannoma. (**A**) Chest CT at the initial presentation revealed a sessile intratracheal mass measuring 11 × 9 × 14 mm. (**B**) Bronchoscopy at the time of biopsy highlighted a broad-based tumor arising from the posterior membranous wall of the trachea and narrowing the tracheal lumen. (**C**) Follow-up CT performed 2 weeks after the initial scan revealed rapid enlargement of the tumor to 17 × 12 × 21 mm, leaving a residual airway diameter of 4.2 mm. (**D**) 3D CT reconstruction (posterior view) illustrating the anatomical relationship between the tumor and the carina.

**Fig. 2:**
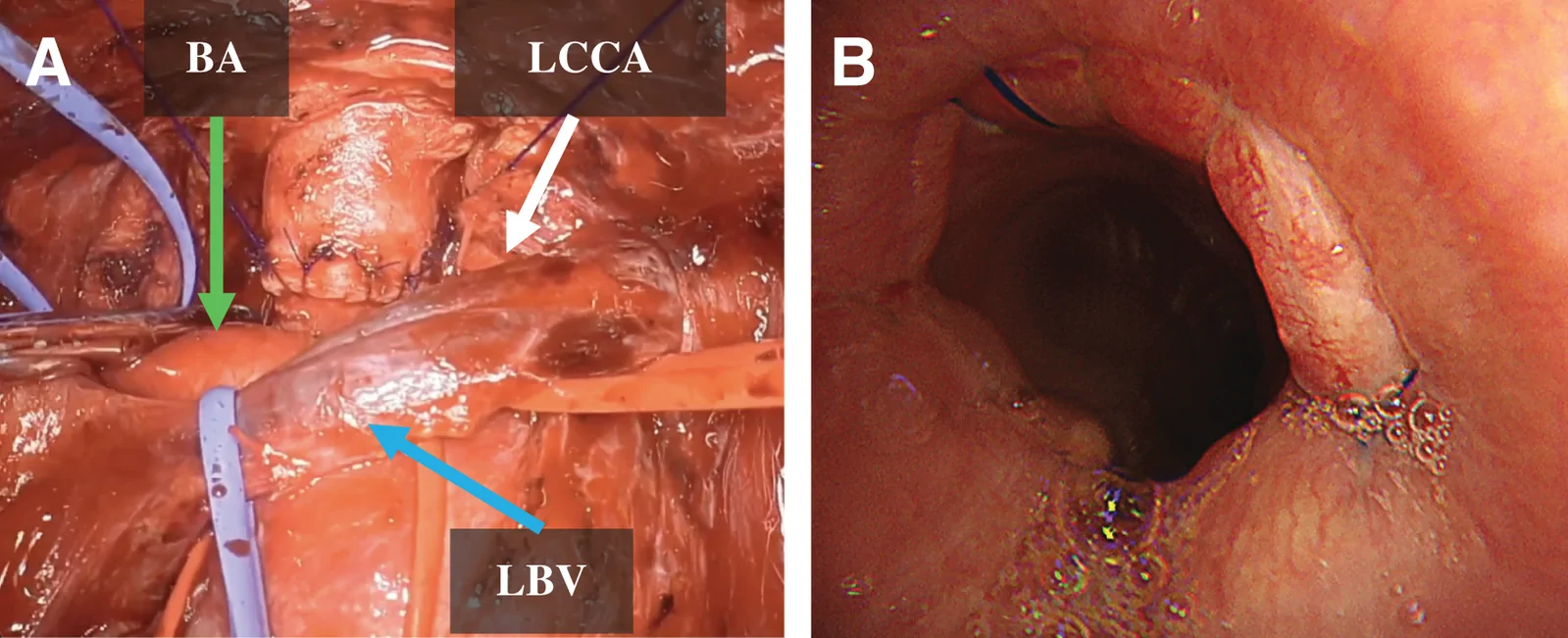
Intraoperative exposure after tracheal reconstruction and postoperative bronchoscopy. (**A**) Intraoperative view via median sternotomy after the completion of end-to-end tracheal reconstruction, revealing exposure of the anterior tracheal wall following mobilization of the BA, LBV, and LCCA. (**B**) Bronchoscopy on POD 9 highlighted an intact anastomosis without dehiscence or stenosis. BA, brachiocephalic artery; LBV, left brachiocephalic vein; LCCA, left common carotid artery

**Fig. 3:**
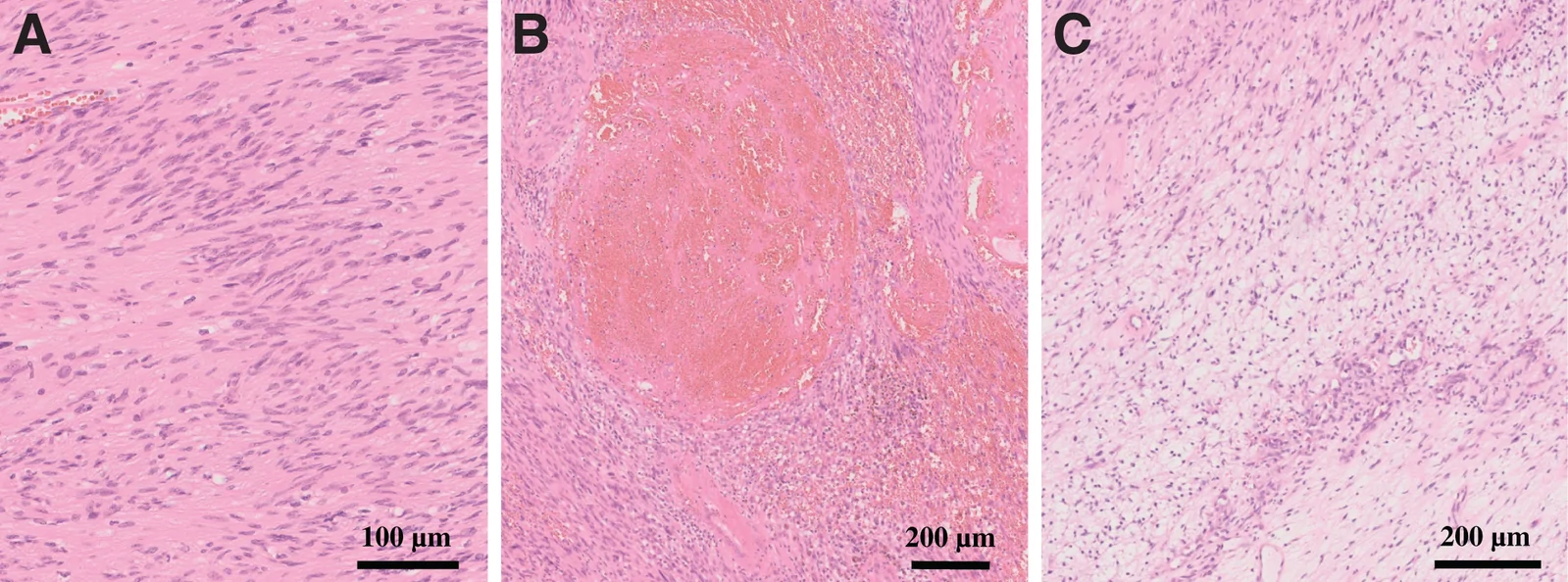
Histopathological findings of the tracheal schwannoma. (**A**) H&E-stained section highlighting spindle cells with nuclear palisading, consistent with benign schwannoma and without histological features of malignancy. (**B**) Intratumoral hemorrhage. (**C**) Inflammatory edema. Scale bars are presented in each panel. H&E, hematoxylin and eosin

**Fig. 4:**
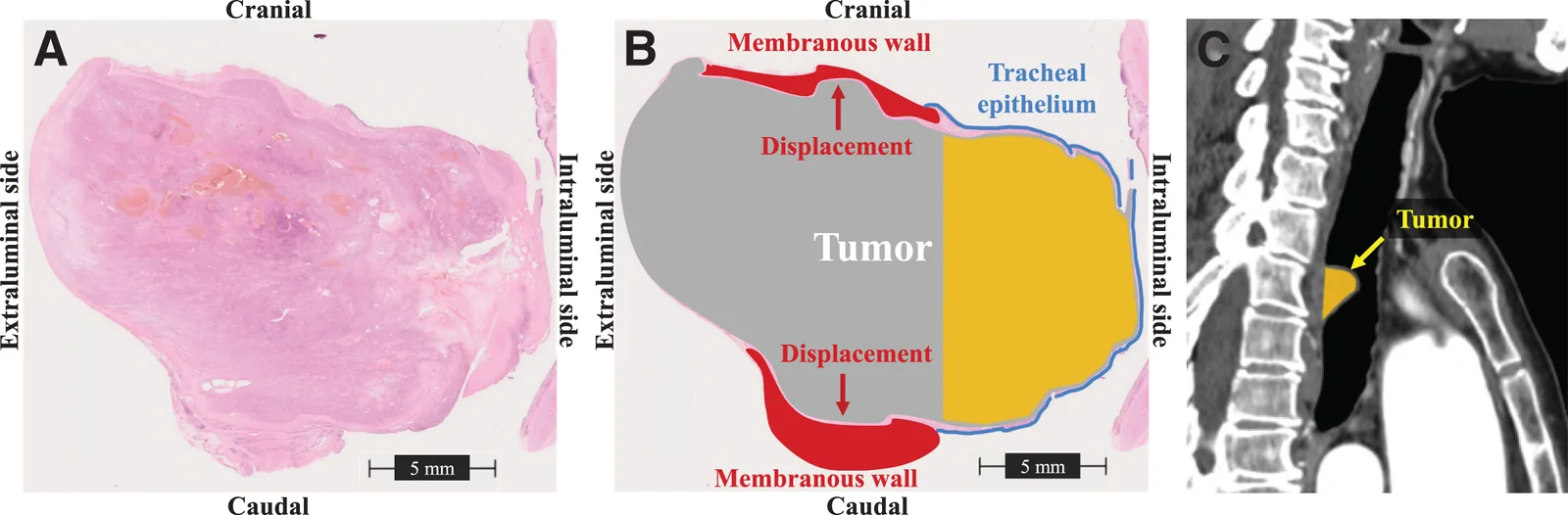
Histological and CT findings of the tracheal schwannoma. (**A**) Low-power sagittal section of the resected tumor. Intraluminal/extraluminal and cranial/caudal orientations are indicated. (**B**) Corresponding annotated low-power photomicrograph showing the relationship between the tumor and adjacent tracheal structures. The tumor is highlighted in gray, and the intraluminal component is highlighted in yellow. The membranous wall is shown in red, and the tracheal epithelium is shown in blue. The tumor exhibited expansile growth, displacing the surrounding tissues, including the membranous wall, and its location suggested an origin from the membranous wall. It extended intraluminally and extraluminally in a dumbbell-shaped configuration. (**C**) Sagittal CT image showing the tumor and its relationship to the tracheal wall in an orientation corresponding to that of the histological section. The intraluminal component of the tumor is highlighted in yellow. Scale bars: 5 mm in panels (**A**) and (**B**).

## DISCUSSION

Primary tracheal tumors comprise an uncommon group of airway neoplasms. Piórek et al. reported an annual incidence of approximately 0.1 per 100000 persons and identified squamous cell carcinoma and adenoid cystic carcinoma as the predominant histologic types.^2)^ Primary tracheal schwannoma is even rarer, accounting for less than 0.5% of primary tracheal tumors.^1)^ Consistent with this rarity, Choi et al. noted that only 52 cases were reported between 1950 and 2015.^3)^ Against this background, the present case represents a rare benign tracheal tumor with an unusual clinical course characterized by rapid preoperative enlargement.

Growth data specific to tracheal schwannomas are limited. Schwannomas generally exhibit indolent growth. The mean growth rate of vestibular schwannomas has been reported as approximately 1 mm per year.^4)^ Conversely, rapid recurrence or enlargement of tracheal schwannomas has occasionally been described. Chen et al. reported a case of transmural tracheal schwannoma that recurred rapidly 12 days after near-complete bronchoscopic resection, ultimately necessitating tracheal resection.^5)^ In their case, the Ki-67 labeling index was 3% in the initial specimen from the bronchoscopic resection, increasing to 20% in the surgical specimen following re-resection. In contrast, in the present case, the Ki-67 labeling index was approximately 15% in both the preoperative biopsy and surgical resection specimens, with no marked increase. Therefore, an increase in proliferative activity following biopsy could not be considered the primary cause of rapid tumor growth in this case. Alternatively, intratumoral hemorrhage has been reported to be associated with rapid volumetric expansion in vestibular schwannoma.^6)^ In the present case, the tumor enlarged within approximately 2 weeks after bronchoscopic biopsy, and the resected specimen displayed intratumoral hemorrhage and inflammation. Therefore, hemorrhage- and inflammation-associated volume expansion may have contributed to the rapid enlargement.

In general, a tracheal tumor with both intraluminal and extraluminal components, producing a dumbbell-shaped configuration, may raise suspicion of transmural malignant extension.^7)^ However, benign tracheal schwannomas can also extend intraluminally and extraluminally, possibly because of the anatomical constraints associated with their origin within the tracheal wall.^8)^ Anatomically, the posterior membranous wall of the trachea contains a complex neural network with contributions from branches of the vagus and recurrent laryngeal nerves.^9)^ Therefore, these neural branches may represent the site of origin of schwannomas arising from the posterior membranous wall. However, in the present case, the nerve of origin could not be precisely determined on pathological examination. Hamouri and Novotny reported a dumbbell-shaped schwannoma arising from the posterior tracheal wall without identifying a parent nerve.^7)^ In the present case, the broad-based attachment made complete bronchoscopic removal difficult. Circumferential tracheal resection was therefore selected to achieve complete tumor removal and permit reliable airway reconstruction. The combined intraluminal and extraluminal configuration identified in the resected specimen further suggested that bronchoscopic treatment alone might have left a residual extraluminal component.

Given the rapid enlargement of the tumor and the residual airway diameter of 4.2 mm, the induction of general anesthesia was considered to carry a substantial risk of ventilation failure or complete airway obstruction. Maxwell and Forrest proposed that planned ECMO support should be considered for airway procedures in patients with severe symptoms caused by subglottic or tracheal obstruction or when the tracheal diameter is less than 5 mm.^10)^ In the present case, conventional airway management was considered to provide an insufficient margin of safety because airway manipulation could have precipitated complete obstruction before rescue ECMO could be established. Therefore, V-V ECMO was established while the patient was awake under local anesthesia, before induction, to maintain oxygenation and facilitate tracheal resection and reconstruction. As the patient was hemodynamically stable and required respiratory support rather than circulatory support, V-V ECMO was selected. Establishing V-V ECMO while the patient was awake secured oxygenation before the induction of general anesthesia and endotracheal intubation, thereby reducing the risk of severe hypoxemia if airway manipulation precipitated complete obstruction. However, ECMO-assisted surgery entails an increased risk of bleeding, particularly when systemic anticoagulation is used. Although no major vascular injury occurred, diffuse chest wall oozing was difficult to control, and the procedure was associated with substantial blood loss and transfusion requirements. The extent to which ECMO-related anticoagulation contributed to the observed bleeding could not be determined. Nevertheless, this experience highlights the trade-off between securing oxygenation and increasing hemorrhagic risk. Therefore, planned ECMO support should be considered when the anticipated risk of ventilation failure or complete airway obstruction outweighs the associated bleeding risk.

## CONCLUSIONS

We encountered a case of benign primary endotracheal schwannoma that exhibited rapid preoperative enlargement. The lesion was resected after V-V ECMO was established before the induction of general anesthesia. Even when a diagnosis of benign schwannoma is suggested, the possibility of rapid enlargement and critical airway compromise should be considered. Establishing V-V ECMO before induction could represent a valuable option when rapid tumor enlargement and critical residual airway narrowing make conventional airway management unreliable.

## DECLARATIONS

### Use of artificial intelligence tools

ChatGPT (OpenAI, San Francisco, CA, USA) was used solely to assist with English-language editing and refinement. It was not used for data collection or analysis, scientific or clinical interpretation, or image generation or modification. All AI-assisted content was critically reviewed and revised by the authors, who take full responsibility for the final manuscript.

### Availability of data and materials

All data supporting the findings of this case report are included in this article.
